# Experimental and computational approaches of methoxy naphthylbithiophene derivatives and their use as corrosion protection for carbon steel in acidic medium

**DOI:** 10.1038/s41598-023-35498-6

**Published:** 2023-05-29

**Authors:** Abd El-Aziz S. Fouda, Safaa-Eldin H. Etaiw, Mohamed A. Ismail, Dina M. Abd El-Aziz, Mohamed M. Eladl

**Affiliations:** 1 Department of Chemistry, Faculty of Science, Mansoura University, Mansoura, 35516 Egypt; 2 Department of Chemistry, Faculty of Science, Tanta University, Tanta, 31527 Egypt; 3 Quality Control Laboratory, Zohr Gas Field, Belayim Petroleum Company, Cairo, Egypt

**Keywords:** Corrosion, Theory and computation

## Abstract

The inhibition efficiency and adsorption affinity were investigated for two novel compounds, namely: 6-methoxy-2-naphthyl-[2, 2’-bithiophene]-5-carboxamidine hydrochloride salt (MA-1440) and 5'-(4-chlorophenyl)-2, 2’-bifuran-5-carboxamidine hydrochloride salt (MA-1456). The inhibition study was conducted on carbon steel surface in 1.0 M HCl with different inhibitor doses and different temperature levels, to investigate the optimum dose and preferable temperature. The performed investigation included chemical, electrochemical, instrumental, and quantum computation techniques. A chemical technique was accomplished by using weight-loss measurements. Different factors were studied using weight-loss measurements in order to reach the maximum inhibition efficiency. The adsorption study revealed that the examined inhibitors obey the Langmuir adsorption isotherm and are chemically adsorbed on the steel surface. The electrochemical measurements were accomplished through the electrochemical impedance (EIS) and potentiodynamic polarization (PDP) techniques. Based on the electrochemical measurements, the examined compounds were categorized as mixed inhibitors. The instrumental examination using different techniques namely: scanning electron microscope (SEM), energy dispersive X-ray (EDX), Fourier-transform infrared spectroscopy (FT-IR), and X-ray photoelectron spectroscopy (XPS) confirmed that the considered inhibitors are excellently adsorbed over the carbon steel surface. The extent of the adsorption affinity of these compounds on the carbon steel surface was studied theoretically using quantum computations and Monte Carlo simulation. The theoretical investigation results of quantum chemistry were validated with those obtained by chemical and electrochemical methodologies. All investigations prove that, the tested compounds were adsorbed chemically on the steel surface and achieved maximum inhibition efficiency of, 94.69% and 90.85% for M-1440 and MA-1456, respectively, at the optimum concentration 30 $\times$ 10^–6^ mol L^−1^ and temperature 328 K.

## Introduction

Acids are widely used in various chemical industries and technological processes in industry, e.g. in pickling baths, in construction of power machine, in heat engineering to remove scales and other deposits from heat-exchange apparatus, in atomic-power installation to deactivate equipment, in the extraction and processing of oil and gas, in rocket technology and in various processes of the chemical and petrochemical industries, etc. Furthermore, the acid can be by produced in chemical processes such as the thermal cracking of petroleum as a result of the hydrolysis of salts and may have a destructive effect on the equipment. To retard the corrosion of materials during the industrial processes, corrosion inhibitors are generally added into the acid solutions. Several types of inhibitors are used to prevent the corrosion in all the industry sectors. Organic inhibitors have been known as effective acid-inhibitors for carbon-steel^1–5^. In general, the action of organic inhibitors functions by forming an adsorbed film on the surface of the corroded metal and its adsorption process and efficiency mainly depend on some molecular structure features of organic molecules and their affinity to be adsorbed on the metal surface. The adsorption can occur via chemical or physical interactions^6^. The adsorbed layer forms a large reaction barrier to the cathodic and anodic processes to occur on metal surface. Organic inhibitors are classified as cationic or anionic or of mixed-type based on their functionality^7^. Large positively-charged aromatic or aliphatic compounds with amine groups usually participate as active portion in organic cationic inhibitors^8^. Heterogeneous organic compounds that exhibit higher basicity and electron density in heteroatoms, such as N, O, and S, tend to retard corrosion. Nitrogen and oxygen are the active centres of the adsorption process on the metal surface. These compounds can be adsorbed on the metal surface, blocking surface-active sites and thus reducing corrosive attack. The effectiveness of these compounds as corrosion inhibitors can be assigned to the number of lone pairs present, the p-orbital character of the free electrons, and the electron density around the nitrogen and oxygen atoms^9–11^. Many of these organic inhibitors have been investigated and found to be appropriate for the prevention of corrosion of steel. Three heterocyclic organic compounds derived from imidazole (IM-OH, IM-OCH_3_, and IM-H) were studied by Ouakki et al.^12^ as corrosion inhibitors for mild steel in a sulfuric acid environment and demonstrated inhibition efficiencies of 97.7%, 98.9%, and 88.9%, respectively at 10^−3^ M. In 1 M HCl, the effects of three heterocycle based on quinoxaline, namely 6-methyl-2,3-diphenylquinoxaline (Q-CH_3_), 6-nitro-2,3-diphenylquinoxaline (Q-NO_2_), and 2,3-diphenyl quinoxaline (Q-H), were examined as mild steel corrosion inhibitors^13^. The inhibitory efficacy of Q-H, Q-CH_3_, and Q-NO_2_ were in the following order: 87.6% (QNO_2_), 90.2% (Q-CH_3_), and 92.4% (Q-H). Three methoxy-substituted phenylthienyl benzamidine derivatives^14^ namely: monocationic 2,5-diarylthiophenes namely; 2-(4-amidinophenyl)-5-(4-methoxyphenyl) thiophene hydrochloride salt (MA-1313), 2-(4-amidinophenyl)-5-(3,5-dimethoxyphenyl)thiophene hydrochloride salt (MA-1314), 2-(4-amidino-3-fluorophenyl)-5-(3,5-dimethoxyphenyl)thiophene hydrochloride salt (MA-1216) were utilized as corrosion inhibitors for carbon steel in 1MHCl.The inhibition efficacy of MA-1313 reaches to 95% at 21 × 10^−6^ M. (2-(4-chlorophenyl)-3-hydroxy-8-styryl-pyrimido [2,1-b][1,3] thiazine-4,6-dione(CSPTD)) as effective inhibitor of mild steel corrosion in HCl solution was studied by Galai et al.^15^. It gave 91.5% inhibition efficacy at 10^–3^ M. El-Faham et al.^16^ reported the effect of 2-hydrazino-4,6-dimethoxy-1,3,5-tirazine (DMeHT), 2,4-dihydrazino-6-methoxy-1,3,5-triaizine (DHMeT), and 2,4,6-tridydrazino-1,3,5-triaizne (TH3) as corrosion inhibitors for steel corrosion in acidic media. The inhibition efficiency was found to be 97.8%, 95.2%, 97.8% forTH3, DMeHT and DHMeT, respectively. Lukman et al.^17^ investigated the suppression of mild steel corrosion in 1 M HCl using 1-[3-phenyl-5-quinoxalin-6-yl-4,5-dihydropyrazol-1-yl] butan-1-one (PQDPB), 1-(3-phenyl-5-(quinoxalin-6-yl)-4,5-dihydro-1H-pyrazol-1-yl)propan-1-one (PQDPP (PPQDPE). For PQDPB, PQDPP, and PPQDPE, the respective inhibition efficiencies were 90.50%, 93.65%, and 86.8%. The inhibitory capacity of (E)-2-((2,5-dichlorophenyl)diazenyl) naphthalen-1-ol (DPD) against mild steel corrosion in 0.5 M HCl has been reported by Amoko et al.^18^ and it yields 76% inhibition protection at 15.8 × 10^–4^ M. Bhandari^19^utilized 4-amino-3-hydroxy-naphthalene-1-sulfonic acid (ANSA) as inhibitor for iron corrosion in 1 M HCl solution and obtained 70% inhibition activity at 70 mg L^−1^. Two newly synthesised Quinoline derivatives, namely 5-((2-(4-dimethylamino)phenyl-1H-benzo[d]imidazol-1-yl)methyl)quinolin-8-ol, Q-N(CH_3_)_2_, and 5-((2-(4-nitrophenyl)-1H-benzo[d]imidazol-1-yl)methyl)quinolin-8-ol, (Q-NO_2_), were studied as inhibitors for the corrosion of mild steel in 0.5 M sulphuric acid solution by Dkhireche et al.^20^. The inhibition efficacy was found to be 93.6% and92.3% for Q-N(CH_3_)_2_ and (Q-NO_2_), respectively. Galai et al.^21^ synthesized, characterized 5-(ethoxymethyl)-8-quinolinol (M-QN), and utilized it as anticorrosion agent for carbon steel in 1.0 M hydrochloric acid solution. Its inhibitive action against the corrosion reached 97.7% at 10^–3^ M additive concentration. Effect of silicon and phosphorus contents in steel on its corrosion inhibition in 5 M HCl solution in the presence of Cetyltrimethylammonium/KI was studied by El Kacimi et al.^22^. The inhibition efficacy for the three classes of steel was found to be 95%, 91% and 77% for classes A, B and C, respectively. The anticorrosive performance of two acrylic polymers (PANa) and (PAA) on mild steel in the 0.1 M HCl medium was investigated by Ouass et al.^23^. The inhibition productivity has attained 97% for PAA and 93% for PANa at5 × 10^−5^ M. 3-methyl-2-(p-tolyl) quinazolin-4(3H)-one (QZ-CH_3_), 2-(4-hydroxyphenyl)-3-methylquinazolin-4(3H)-one (QZ-OH), 3-methyl-2-(4-nitrophenyl) quinazolin-4(3H)-one (QZ-NO_2_) and 3-methyl-2-phenylquinazolin-4(3H)-one (QZ-H) were synthesized, characterized and utilized as inhibitors for against mild steel corrosion in 1.0 M HCl^24^, their inhibition efficacies reached maximum values of 92%, 96%, 82%, and 81% at 10−3 M of QZ-OH, QZ-CH_3_, QZ-NO_2_ and QZ-H, respectively.

However, with increasing environmental awareness, emphasis is being placed on using appropriate inhibitors that are non-toxic or less toxic^25^.

The novelty of this work is that methoxy naphthybithiophene and 4-chlorophenylbifuran derivatives are heterocyclic aromatic organic compounds that contain electron donating groups and π electrons, which can induce greater adsorption and weaken corrosion in the C-steel surface. Moreover, in recent studies the compounds exhibit antioxidant and antiproliferative activity against several cancer cells without causing any toxicity in normal cells ^26^ were used as corrosion inhibitors.

The goal of the current experiment was to assess 6-methoxy's ability to suppress corrosion. 2-naphthyl-[2, 2'-bithiophene] 5-carboxamidine hydrochloride salt (MA-1440) and five'- (4-chlorophenyl) -2, 2'-bifuran-5-carboxamidine hydrochloride salt (MA-1456) using chemical *(WL)* and electrochemical *(PDP, EIS)* techniques of monitoring corrosion and characterizing the surface morphology of the corroded steel specimens submerged in the test corrosive medium in both the absence and presence of the additives. Also, quantum chemical calculations and molecular dynamics replication have been examined and discussed.

## Materials and procedures

### Inhibitors

**Figure 1:**
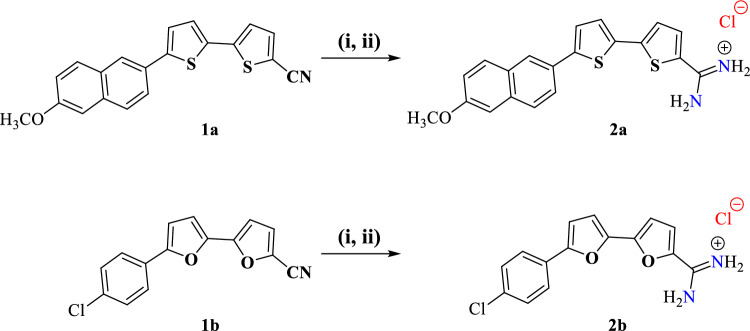
Synthesis scheme of the studied 6-methoxy-2-naphthyl-[2, 2’-bithiophene]-5-carboxamidine (**2a**) and 5'-(4-chlorophenyl)-2, 2’-bifuran-5-carboxamidine (**2b**). Reagents and conditions: (i) LiN (TMS)_2_ r.t. (ii) ethanol/hydrogen chloride, r.t.

The investigated compounds namely 6-methoxy-2-naphthyl-[2,2’-bithiophene]-5-carboxamidine and 5'-(4-chlorophenyl)-2,2’-bifuran-5-carboxamidine inhibitors were synthesized previously and characterized as recently reported by Ismail et al.^26^. Figure 1 and Table 1 illustrate the molecular structures and the formulas of these compounds. The structure of both inhibitors has the same counter ion Cl^−^, thus it can be assumed that the effect of this counter ion is negligible in dominant acidic solution of 1.0 M HCl. The investigated compounds were received pre-synthesized in powder form. In order to study the effect of these compounds as corrosion inhibitors, stock solutions 0.001 mol L^−1^ were prepared. The equivalent amount of inhibitor was dissolved in 10 ml dimethyl-sulfooxide then the volume was completed up to 100 ml with absolute ethanol. The studied inhibitors were used at different concentrations (5–30 $\times$ 10^–6^) mol L^−1^ by dilution from the stock solution.

**Table 1:** Chemical structure, nomenclature, molecular formula and formula weights (F) of the examined compounds.

Code	Molecular structures/Name	Molecular formulas, (F. wt.)
MA-1440	6-Methoxy-2-naphthyl-[2,2’-bithiophene]-5-carboxamidine hydrochloride salt	C_20_H_16_N_2_OS_2_–1.0HCl–1.5H_2_O (427.98)
MA-1456	5'-(4-Chlorophenyl)-2,2’-bifuran-5-carboxamidine hydrochloride salt	C_15_H_11_ClN_2_O_2_–1.0HCl–0.8H_2_O (337.57)

### Corrosive media

Hydrochloric acid was used as a corrosive solution with concentration 1.0 mol L^−1^. Analytical grade 37% HCl solution was used for freshly preparation of 1.0 mol L^−1^ HCl by dilution with ultra-pure demineralized water.

### Carbon steel specimens

API 5L X70 C-steel sheets (chemical composition: C max 0.28%, Mn max 1.40%, P max 0.030%, S max 0.030%, Ni max 0.50%, Gr max 0.50%, Cu max 0.50%. The sum of the Nb, V and Ti ≤ 0.06% and the balance as Fe) were used in implementing the experiments and the measurements. C-steel coupons were cut out from the sheets with dimensions: 2 cm × 2 cm × 0.2 cm. The coupons were firstly de-rusted and polished with different grades of ISO/FEPA Grit sandpapers (P220–P2000) until a mirror surface was observed.

### Weight-loss *(WL)* method

WL measurement is a common and preferred chemical approach used on laboratory scale to measure the corrosion rate and hence the efficiency of corrosion inhibitors. C-steel coupons were subjected to de-rusting and polishing as explained above in Sect. 1.3. The weight of coupons was recorded before dipping in the test solution and after 30 min from dipping. The difference between the initial weight of the coupons and the weight after 30 min of acid exposure was considered the WL which was used to calculate the rate of corrosion and inhibition efficiency for C-steel. The measurements of WL were measured each 30 min over 6 h., run at different temperatures (298, 308, 318 and 328 K). The surface coverage ($\uptheta$), corrosion rate (CR) and inhibitor efficiency ($\eta$%) were estimated using the following equations^27^.

1 $$CR=\frac{W}{A t}$$

2 $$\theta = \frac{CR-{CR}_{(i)}}{CR}$$

3 $$\eta \%= \theta x100$$

where W is the carbon steel WL in mg, *CR*_*(i)*_ and *CR* express the corrosion rates of coupons in presence and absence of the inhibitors, A is the exposed surface area of the coupon in cm^2^ and t is the dipping time in h.

### Electrochemical measurements

The measurements were conducted in the three- electrodes electrochemical cell by Gamry with reference 3000 Potentiostat/Galvanostat/ZRA analyser. The working electrode was fabricated from carbon steel (API 5L X70) with dimensions 1 cm × 1 cm × 0.2 cm and welded from one side with a copper rod covered with a glass pipe. A saturated calomel electrode (SCE) and a platinum wire were used as the reference electrode and the auxiliary electrode, respectively. The experiments were performed in HCl solution (1.0 M) in the absence and presence of different doses of inhibitors at temperature 298 K. The working electrode was immersed firstly in the solution under testing for 30 min to reach the steady state potential, Open Circuit Potential (OCP). The efficiency of inhibitor was estimated from the electrochemical cell using two approaches, Potentiodynamic Polarization (PDP) and Electrochemical Impedance Spectroscopy (EIS). The corrosion current density was measured from the Potentiodynamic Polarization under steady state conditions while applying potentials ranging from − 250 to + 250 mV against OCP at a rate of 1 mV/s. The Potentiostat device recorded and plotted the relationship between the acquired corrosion current density and the Tafel polarization. The Electrochemical Impedance was measured at OCP using a multi-frequency AC electrochemical approach with signal amplitude of 10 mV and a frequency range from 100 kHz to 0.2 Hz. The attained EIS data give the opportunity to investigate polarization resistance, high-frequency solution resistance, and double layer capacitance (low-frequency region). Typically, polarization resistance is used to calculate the corrosion rate^28^. The Nyquist and Bode plots were used to represent the obtained EIS results.

#### Surface morphology studies

### Scanning electron microscopy (SEM)/energy dispersive X-ray (EDX) analysis

To investigate the surface morphology and elemental composition of glossy and corroded C-steel with and without applying inhibitors, as well as to determine the surface elemental composition, scanning electron microscopy (SEM) model JEOL-JSM-6510LV, Japan, and energy dispersive X-ray (EDX) were used. The samples were submerged in 1.0 M HCl for 6 h. in non-presence and presence of the inhibitors at the optimal dose. Before inspection, samples were dried at room temperature.

### Fourier-transform infrared spectroscopy (FT-IR) analysis

Thermo Fisher Scientific, USA, Nicolet iS10 spectrophotometer was used to produce the IR spectra in the mid-IR range of 400–4000 cm^−1^ with a spectral resolution of 4 cm^−1^ and 128 scans. To compare and demonstrate the adsorption of inhibitors on the C-steel surface, FT-IR analysis was performed for the retrieved specimens from test solution after immersion for 6 h. performed for the pure inhibitors versus Attenuated Total Reflectance IR spectroscopy (ATR-IR).

### X-ray photoelectron spectroscopy (XPS) analysis

The elemental composition of the specimen's surface was inspected using the XPS technique, to provide evidence about the nature of the adsorption process of the inhibitive layer on the C-steel surface. In this way the type of chemical bonding on the surface of carbon steel was recorded. XPS was performed using a Thermo Fisher Scientific, USA, model K-alpha with X-ray monochromatic Al K-alpha radiation ranging from − 10 to 1350 eV, at pressures 9 and 10 mbar, with a spot size of 400 m, 200 eV of full spectrum pass energy and 50 eV of narrow spectrum pass energy.

#### Computational methods

### Quantum chemical calculations

Based on various methods, the computations from quantum chemical theories were employed as a useful tool for understanding the material properties and the corrosion inhibition process^29,30^. In Material Studio software (version 7.0), the Density Functions Theory (DFT) was implemented utilizing the Dmol3 module to investigate the full geometrical optimization of the inhibitors under study. The molecular and electronic structures of the two derivatives express the efficiency as well as their corrosion inhibition behaviour. The DFT technique was used to estimate the quantum parameters, including the energies of the highest occupied (HOMO) and lowest unoccupied (LUMO) molecular orbitals, electronegativity *(χ)*, dipole moment, chemical potential *(μ),* smoothing *(σ)* and absolute hardness *(η).*

### Monte-Carlo simulations

Through the Monte Carlo simulations, the optimal arrangement of the examined compounds on the surface of Fe (110) was evaluated. According to the literature^31^, the Fe (110) crystal surface is assumed to be the most stable surface used in this simulation. To simulate the solvent effect during the corrosion process, 100 water molecules were applied to study the adsorption of uncharged and protonated inhibitor molecules. The quotation module was used to perform geometrical optimization of water and inhibitor molecule firstly. By doing a Monte-Carlo search of the configuration space for the substrate-adsorbate system, adsorption sites with low energy were identified, where the temperature is gradually decreased. The Monte-Carlo simulation was used to determine the total energy, adsorption energy, and the energy of substrate-adsorbate configurations.

## Results and discussion

### Weight-Loss *(WL)* method

#### Impact of concentration

**Figure 2:**
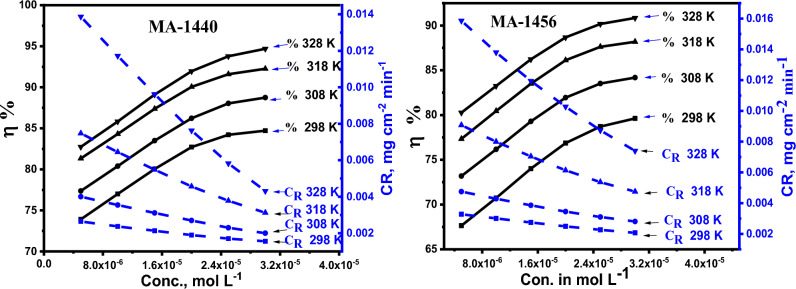
Impact of different concentrations of inhibitors MA-1440 and MA-1456 on CR and *η%* on the corrosion of C-steel in inhibited test solutions at different temperatures.

Different inhibitor concentrations in the range of 5–30 10^–6^ mol L^−1^ were selected to evaluate the effect of inhibitor concentration on corrosion behaviour. To assess the rate of corrosion *(CR)* and effectiveness of the inhibitor $(\eta \%$*)*, C-steel was submerged in 1.0 M HCl solution for six hours. Figure 2 and Table 2 show a considerable reduction in the CR of C-steel as well as an improvement in inhibition effectiveness as a result of increasing inhibitor concentration. This can be explained by the fact that increasing the amount of inhibitor leads to an increase in the surface area coverage of C-steel, improves inhibitor adsorption on the metal surface and inhibits the corrosion^32,33^. As a result, the lowest *CR* and best $\eta \%$ are observed at the maximum concentration. According to Fig. 2, it is evident that the *CR* and $\eta \%$ curves reach a plateau at the concentration of 30 × 10^−6^ mol L^−1^ of added inhibitor in solution, which represents the optimized quantity to obtain the maximum benefit with the lowest possible concentration. Therefore, it is worthless to increase the inhibitor amount more than this optimal dose. The maximum $\eta \mathrm{\%}$ reaches 94.69% and 90.85% for MA-1440 and MA-1456, respectively. The difference in efficiency of two investigated inhibitors is related to the molecular structure of these derivatives.

**Table 2:** Corrosion criterions from *WL* for C-steel dissolution in 1.0 M HCl in the presence and absence of inhibitors at the studied temperatures.

Inhibitor	Conc., M	298 K		308 K		318 K		328 K	
		*CR* × 10^−3^ mg cm^−2^ min^−1^	*ɳ%*	*CR* × 10^−3^ mg cm^−2^ min^−1^	*ɳ%*	*CR* × 10^−3^ mg cm^−2^ min^−1^	*ɳ%*	*CR* × 10^−3^ mg cm^−2^ min^−1^	*ɳ%*
MA-1440	1.0 M HCl	10.1	–	17.7	–	40	–	80.4	–
	5 × 10^−6^	2.64	73.91	4.00	77.37	7.47	81.33	13.87	82.74
	10 × 10^−6^	2.37	77.00	3.54	80.39	6.45	84.31	11.72	85.83
	15 × 10^−6^	2.13	80.04	3.11	83.50	5.48	87.38	9.62	89.11
	20 × 10^−6^	1.90	82.71	2.69	86.22	4.56	90.05	7.630	91.96
	25 × 10^−6^	1.70	84.21	2.30	88.04	3.78	91.59	5.83	93.78
	30 × 10^−6^	1.55	84.71	2.00	88.74	3.12	92.25	4.31	94.69
MA-1456	5 × 10^−6^	3.28	67.64	4.75	73.18	9.07	77.33	15.86	80.27
	10 × 10^−6^	3.01	70.73	4.29	76.17	7.99	80.45	13.80	83.25
	15 × 10^−6^	2.74	74.01	3.86	79.29	7.04	83.50	11.94	86.23
	20 × 10^−6^	2.49	76.86	3.45	81.95	6.13	86.12	10.27	88.68
	25 × 10^−6^	2.26	78.71	3.10	83.52	5.37	87.62	8.73	90.17
	30 × 10^−6^	2.07	79.62	2.81	84.19	4.75	88.19	7.40	90.85

#### Impact of temperature

**Figure 3:**
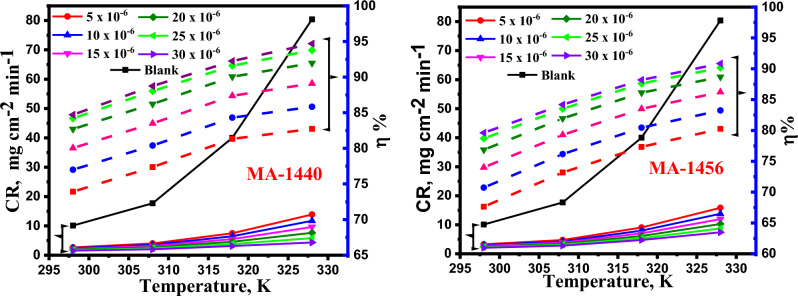
Impact of temperature on *CR* and *η%* on the corrosion of C-steel in inhibited test solutions at different doses of MA-1440 and MA-1456.

*WL* measurements were used to examine the effect of temperature on the corrosion behaviour of C-steel in the absence and presence of varied doses of inhibitors at the temperatures of 298, 308, 318 and 328 K. As illustrated in Fig. 3 and Table 2**,** with increasing the temperature from 298 to 328 K, the *CR* increases slowly in the presence of inhibitors, while it steeply increases in the absence of inhibitors. Additionally, $\eta \%$ enhances with increasing the temperature. This indicates the corrosion inhibition process is maintained to a greater extent with the investigated inhibitors at elevated temperature. This can be explained considering that increase in temperature leads to increase the molecules interactions with the steel surface and, consequently improve the inhibitors adsorption on the C-steel surface^34^. It well-known that, the behaviour of adsorption is associated with temperature effect on the corrosion inhibition^35,36^. This suggests that, the investigated derivatives are chemically adsorbed on the C-steel surface^37,38^. In particular the considered inhibitors can be adsorbed over the metal surface via donor–acceptor interactions between the lone pairs of electrons on the heteroatoms and π-orbitals with the vacant d-orbital of iron atoms on the steel surface. In addition, the replacement of adsorbed H_2_O molecules from the steel-surface with inhibitor molecules is also occurring. These two combined effects consequently decrease the corrosion rate due to the blocking the active sites on the surface^39^.

### Thermodynamic activation parameters

**Figure 4:**
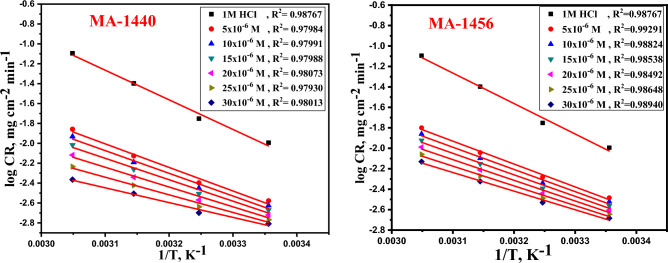
Arrhenius plots of the dissolution of C-steel in 1.0 M HCl with absence and presence of several doses of inhibitors MA-1440 and MA-1456.

**Figure 5:**
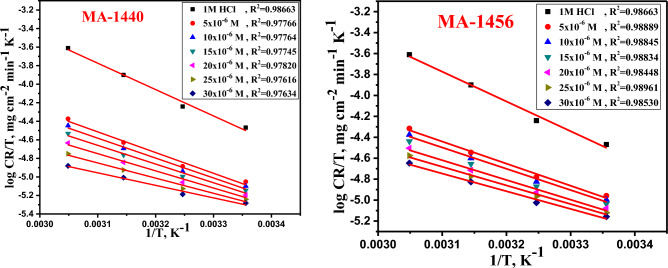
Kinetic transition state plots of the dissolution of C-steel in 1.0 M HCl with absence and presence of several doses of inhibitors MA-1440 and MA-1456.

The thermodynamic activation parameters based on *WL* measurements at temperatures ranging from 298 to328 K can also be used to express the adsorption behaviour of the investigated inhibitors. Using the Arrhenius equation, the effect of temperature on CR can be expressed according to the following equation^40^:

4 $$\mathrm{log} CR=\left(\frac{-{E}_{a}^{*}}{2.303RT}\right)+ log A$$

where *E*_a_^*^ is the apparent activation energy, T is the absolute temperature in kelvin (K), R is the universal constant of gases (8.314 J K^−1^ mol^−1^) and A is the Arrhenius pre-exponential factor. The E^*^_a_ is calculated from the slope, E^*^_a_ =  − (slope) × 2.303R, of the linear plot of log CR against 1/T in Fig. 4. The recorded E^*^_a_ values in Table 3, exhibit a gradual decreasing associated with the increase of inhibitor concentration in the solution. This indicates the adsorption of inhibitors on the metal surface is facilitated with increasing the temperature of the medium, as a result of lowering the energy barrier^41^. Moreover, the decreasing in E^*^_a_ values refers that, the interaction of inhibitor molecule’s with iron atoms on the metal surface follows the chemisorption behaviour^42,43^. Entropy (ΔS^*^) and enthalpy (ΔH^*^) are important thermodynamic parameters, obtained from the transition state equation^44^ as following:

5 $$\mathrm{log}CR/T=\mathrm{log}\left(\frac{\mathrm{R}}{\mathrm{Nh}}\right)+\frac{\Delta {S}^{*}}{2.303\mathrm{R}}-\frac{\Delta {H}^{*}}{2.303\mathrm{RT}}$$

where ∆S^*^ and ∆H^*^ represent the activated entropy and the activated enthalpy, respectively. h is the Planck’s constant (6.6260755 × 10^−34^ J·s) and N is the Avogadro's number (6.02214076 × 10^23^). From the linear fitting of kinetic transition state plots (log *CR/T* against *1/T),* Fig. 5, ∆S^*^ is calculated from the intercept (log *(R/Nh)* + ∆S^*^/2.303R) while ∆H^*^ is calculated from the slope (− ∆H^*^/2.303R). As recorded in Table 3, the positive values of activated enthalpy indicate the endothermic dissolution process^45^, whereas the negative sign of activated entropy assumes that the formation of activated complex is promoted by the reduction in disordering and more tending in the direction of association rather than dissociation in the rate determining step^46,47^.

**Table 3:** Thermodynamic criterions for C-steel dissolution in 1.0 M HCl at several doses of inhibitors calculated from *WL* assessments.

Inhibitor	Conc., M	*E*_a_* kJ mol^−1^	∆H* kJ mol^−1^	− ∆S* J mol^−1^ K^−1^
MA-1440	5 × 10^−6^	45.37	42.74	151.34
	10 × 10^−6^	43.69	41.07	157.83
	15 × 10^−6^	41.23	38.61	166.94
	20 × 10^−6^	38.09	35.47	178.38
	25 × 10^−6^	33.93	31.31	193.20
	30 × 10^−6^	28.46	25.84	212.19
MA-1456	5 × 10^−6^	42.84	39.67	159.51
	10 × 10^−6^	41.59	38.20	165.16
	15 × 10^−6^	40.27	36.78	170.68
	20 × 10^−6^	38.72	35.39	176.17
	25 × 10^−6^	36.92	33.49	183.27
	30 × 10^−6^	34.87	31.76	189.85

### Adsorption isotherm study

**Figure 6:**
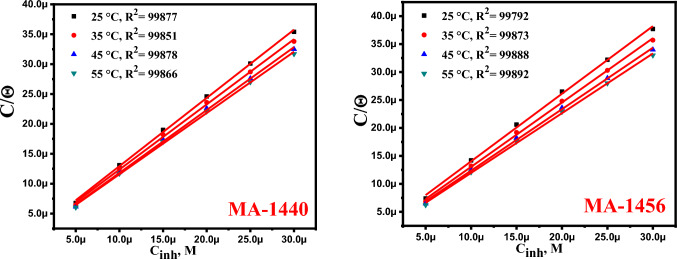
Langmuir isotherm graphs of the carbon steel dissolution in 1.0 M HCl in the presence of MA-1440 and MA-1456 at different temperatures.

Several adsorption isotherms control the interaction between the organic inhibitors and the C-steel surface. Among them, the Frumkin, Langmuir, and Temkin isotherms are the most widely used adsorption isotherms^48^. The fitted experimental data elucidates that the Langmuir isotherm describes properly the interactions of the investigated inhibitors with the metal surface according to the Eq. (6) ^49^:

6 $$\frac{{C}_{inh}}{\theta }=\frac{1}{{K}_{ads}}+ {C}_{inh}$$

where *K*_*ads*_, and C_inh_ symbolize the adsorption equilibrium constant and inhibitor concentration, respectively. Figure 6 elucidates the linear plots of *C*_*inh*_* /θ* against *C*_*inh*_ at different temperatures. The correlation coefficients (R^2^) and slopes of linear plots are close to one, as expected. For both the examined inhibitors, the obtained *K*_*ads*_ values (Table 4) have significant positive values and progressively rise with increasing inhibitor concentrations in the medium, indicating excellent adsorption efficiency and, subsequently, improved corrosion inhibition efficiency^50^. Given the obtained *K*_*ads*_ results, the corresponding values of *ΔG°*_*ads*_ can be calculated using the Eq. (7) ^51^:

7 $${K}_{ads}=\left(\frac{1}{55.5}\right) exp\left(\frac{{-\Delta G}_{ads}^{o}}{RT}\right)$$

where 55.5 is the molar concentration (mol L^−1^) of H_2_O in the solution. As stated in previous^34^, the values of *ΔG°*_*ads*_ give an indication about the type of adsorption mechanism for the inhibitor over the metal surface, which is classified as a physisorption process, if the magnitudes of *ΔG°*_*ads*_ is up to − 20 kJ mol^−1^ or as a chemisorption if the magnitudes are more negative than −40 kJ.mol^−1 52,53^. In our case, the obtained *ΔG*^*°*^_*ads*_ values indicate that the investigated inhibitors are chemically adsorbed on the C-steel surface, and the strength of interaction increases with temperature, as shown in Table 4**,** in which the *ΔG*^*°*^_*ads*_ display large negative values above − 40 kJ mol^−1^ for all inhibitors, and negatively increase with increasing the temperature. The heat of adsorption (*ΔH*^*°*^_*ads*_) and the standard entropy of adsorption (*ΔS*^*o*^_*ads*_) are computed from Van't Hoff and Gibbs–Helmholtz equations^54,55^, respectively:

**Table 4:** The calculated parameters from the Langmuir adsorption isotherms of MA-1440 and MA-1456 inhibitors at different temperatures.

Inhibitor	Temp	*K*_*ads*_ × 10^5^	*−ΔG*^*°*^_*ads*_	*ΔH*^*°*^_*ads*_	*ΔS*^*°*^_*ads*_
	K	M^−1^	kJ mol^−1^	kJ mol^−1^	J mol^−1^ K^−1^
MA-1440	298	6.58	43.15	6.52	166.68
	308	6.79	44.68		145.06
	318	7.77	46.49		146.19
	328	7.54	47.87		145.93
MA-1456	298	5.12	42.53	3.54	154.61
	308	6.47	44.55		144.65
	318	7.21	46.29		145.56
	328	7.77	47.95		146.19

8 $${\mathrm{log }K}_{\mathrm{ads}}=\frac{-\Delta {H}_{ads}^{o}}{2.303\mathrm{RT}}+\frac{\Delta {S}_{ads}^{o}}{2.303R}-\mathrm{log}\left(55.5\right)$$

9 $${\Delta G^\circ }_{ads }= {\Delta H}_{ads}^{o}-T{\Delta S}_{ads}^{o}$$

**Figure 7:**
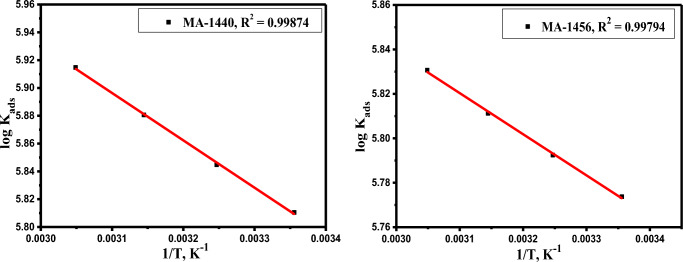
Log *K*_*ads*_ versus 1/T for MA-1440 and MA-1456.

Figure 7 shows the liner plots of log *K*_*ads*_ against 1/T. The heat of adsorption (*ΔH*^*°*^_*ads*_) and the standard entropy of adsorption (*ΔS*^*o*^_*ads*_*)* are calculated from the slopes and intercepts of the fitted plots, respectively, in Table 4. The positive values of *ΔH°*_*ads*_ denote the endothermic interaction, which in turn reflects the chemisorption behaviour of the inhibitors on the steel surface^56^, while the positive *ΔS*^*o*^_*ads*_ values indicate that randomness is increasing at the metal inhibitor interface as a result of water desorption from the C-steel surface during the interaction process^57^.

### Electrochemical studies

#### Potentiodynamic polarization (PDP) studies

**Figure 8:**
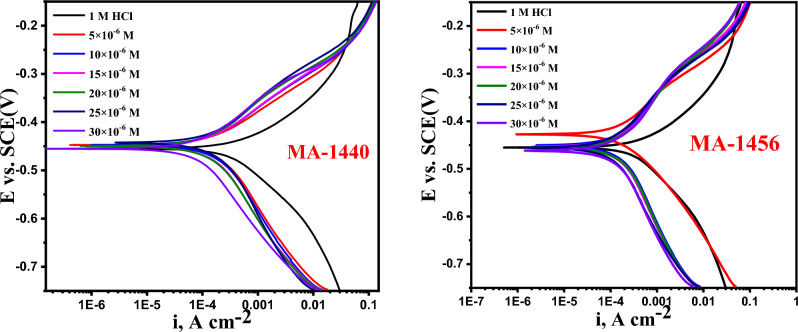
PDP graphs of the C-steel dissolution in 1.0 M HCl with absence and presence of different doses of MA-1440 and MA-1456 at 298 K.

Figure 8 elucidates the anodic and cathodic polarization Tafel plots for the dissolution of the C-steel electrode in 1.0 M HCl in absence and presence of different concentrations from the studied inhibitors at 298 K. After the addition of inhibitors to 1 M HCl, it is evident from Fig. 8 that both the anodic and cathodic curves switched in the direction of lower corrosion current, indicating the mixed nature of the inhibitors. The shape of anodic curves revealed the possibility of formation of complex on the metal surface. The corrosion current density (i_corr_) can be calculated from the extrapolation of linear regions of Tafel plots. Given the obtained electrochemical kinetic variables, the inhibition performance *(%η*_*PDP*_*)* of the investigated inhibitors is calculated as follows^58^:

10 $$\%{\eta }_{PDP}=\frac{{i}_{corr}- {i}_{corr}^{o}}{{i}_{corr}} \times 100$$

where, *i*^*o*^_*corr*_ and *i*_*corr*_ symbolize the density of corrosion current of the C-steel dissolution in 1.0 M HCl with and without inhibitors, respectively. When compared with the blank solution (1.0 M HCl without inhibitor), the obtained data for inhibitors (Table 5) show a significant reduction in the corrosion current density *(i*_*corr*_*),* and the decrease continues as the inhibitor concentration is increased indicating that inhibitor molecules have adhered to the metal surface and has the effect of raising the %IE. According to the Tafel values for the anodic and cathodic slopes *(β*_*a*_*, β*_*c*_*),* both investigated inhibitors act as a mixed type, in which the addition of inhibitors to an acidic solution affects both the anodic and cathodic branches of the curves slightly^59^. Furthermore, the corrosion potentials in inhibitor-containing solutions are similar to those in the blank solution, with a slight shift detected in Tafel curves for the cathodic area. The inhibitor is often categorised as anodic or cathodic if the E_corr_ difference between the blank and the inhibitor-contained solutions is larger than 85 mV, and as mixed type if the difference is less than 85 mV^60,61^, as in the current study. As illustrated by the obtained *E*_*corr*_ values, the maximum shift is 23 mV in the case of MA-1440 inhibitor and 33 mV for MA-1456 inhibitor, so these derivatives act as mixed type inhibitors. The interpreted data demonstrates that the inhibition performance increases with increasing the inhibitor dose in the test solution because a non-conducting layer is formed, acting as a physical barrier in protecting the metal from dissolution in the medium^62^. The %IE of these derivatives is MA-1440 > MA-1456.

**Table 5:** The obtained parameters from PDP studies for the C-steel dissolution in 1.0 M HCl with absence and presence of various doses of MA-1440 and MA-1456 inhibitors at 298 K.

Inhibitors	PDP data						
	M Mol L^−1^	− E_corr,_ mV vs. SCE	i_corr_ μA m^−2^	− β_c_ mVdec^−1^	β_a_ mVdec^−1^	θ	%η_PDP_
	Blank	454.7 ± 0.2028	636 ± 0.1453	245.5 ± 0.2309	153.5 ± 0.1522	–	–
MA-1440	5 × 10^–6^	446.8 ± 0.1553	174.8 ± 0.1732	213 ± 0.1453	121 ± 0.1752	0.725	72.5
	10 × 10^–6^	447.4 ± 0.1653	153.9 ± 0.2309	182 ± 0.1653	113 ± 0.1202	0.758	75.8
	15 × 10^–6^	449.9 ± 0.1453	145.6 ± 0.2082	174 ± 0.1553	93.6 ± 0.2603	0.771	77.1
	20 × 10^–6^	450.5 ± 0.2055	129.7 ± 0.2123	182 ± 0.1732	86 ± 0.1453	0.796	79.6
	25 × 10^–6^	443.1 ± 0.1452	118.1 ± 0.2234	168 ± 0.2309	78.1 ± 0.2309	0.814	81.4
	30 × 10^–6^	454.3 ± 0.2102	106.8 ± 0.2131	156 ± 0.2028	74 ± 0.1732	0.832	83.2
MA-1456	5 × 10^–6^	428.2 ± 0.1353	210.3 ± 0.1732	121 ± 0.2309	82 ± 0.2028	0.669	66.9
	10 × 10^–6^	450.6 ± 0.1725	192.1 ± 0.2309	217 ± 0.1732	101.1 ± 0.1732	0.698	69.8
	15 × 10^–6^	453.5 ± 0.2333	169.6 ± 0.2028	210.4 ± 0.1523	134.5 ± 0.1553	0.733	73.3
	20 × 10^–6^	454.6 ± 0.2121	151.3 ± 0.1453	207.8 ± 0.1453	101.2 ± 0.3528	0.762	76.2
	25 × 10^–6^	455.5 ± 0.2028	140.8 ± 0.1453	199 ± 0.1202	121 ± 0.3528	0.779	77.9
	30 × 10^–6^	461.3 ± 0.2228	134.6 ± 0.2028	191.9 ± 0.2028	109.8 ± 0.3528	0.788	78.8

### Electrochemical impedance spectroscopic (EIS) studies

**Figure 9:**
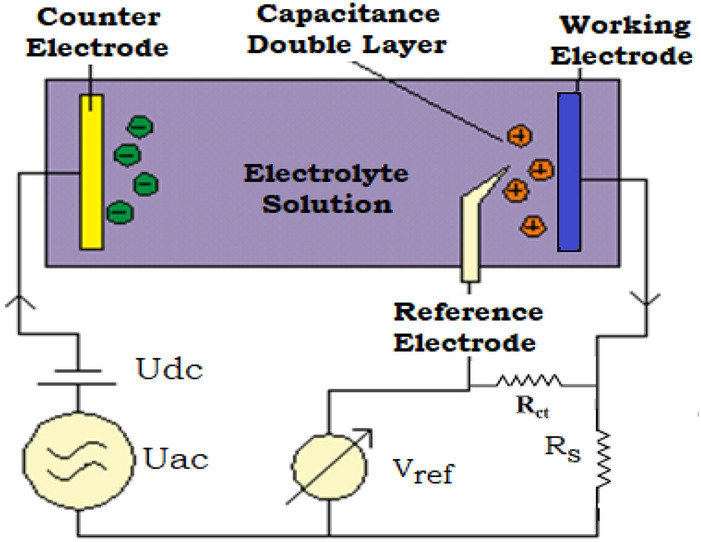
Schematic electrical circuit utilized for investigate EIS statistics.

**Figure 10:**
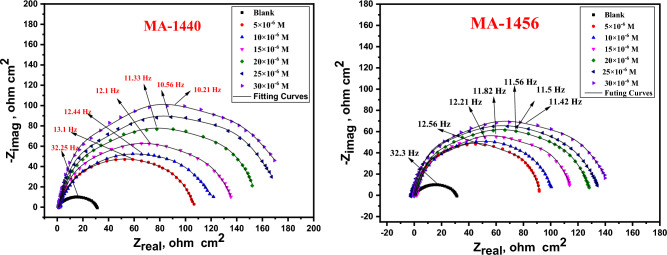
Nyquist graphs of the carbon steel dissolution in 1.0 M HCl with absence and presence of different doses of MA-1440 and MA-1456 inhibitors at 298 K.

The electrochemical impedance measurements were used to track the inhibition behaviour for the investigated inhibitors in corrosive conditions. The electrical characteristics derived from the electrical equivalent circuits are typically used to describe the electrochemical impedance results using the Nyquist and Bode plots. The measurements were conducted in the absence and presence of different doses of inhibitors in 1.0 M HCl at 298 K. The schematic diagram of the EIS equivalent circuit is shown in Fig. 9, where *R*_*s,*_* R*_*c*t_ and *C*_*dl*_ symbolize the resistance of the test solution, the charge transfer, and the double layer capacitance, respectively. Figure 10 illustrates the semicircles Nyquist graphs that arise from the graphic representation of the real part of the impedance versus the imaginary one. As evidently displayed from the Nyquist graphs, all the spectra exhibit the same pattern, confirming the occurrence of corrosion by the same mechanism. However, the width of the semi-circular curves growths with the rise in the concentration of the inhibitor in the solution, indicating an increase in the impedance due to the adsorption of the inhibitor molecules over the surface of the C-steel, which increases the resistance of metal dissolution in the medium^63^. The impedance parameters of C-steel in 1.0 M HCl are listed in Table 6 including double layer capacitance *(C*_*dl*_*),* charge transfer resistance *(R*_*ct*_*)* and inhibition efficiency *(%η*_*EIS*_*).* Given the charge transfer resistance, the inhibition efficiency is calculated using the following equation^64^:

11 $${\%IE}_{EIS}=\frac{{R}_{ct}-{{R}^{*}}_{ct}}{{R}_{ct}}\times 100$$

where R*ct and Rct are the resistances of the charge-transfers in the absence and presence of inhibitors, respectively. As illustrated, the increase in Rct (which is inversely proportional to CR) is associated with the increase in inhibitor efficiency proportional to the inhibitor dose used. Conversely, the double layer capacitance (Cdl) gradually decreases as the inhibitor concentration increases. This is owing to the continuous displacements of water molecules adsorbed from the steel surface with the inhibitor molecules and therefore to the increase in the thickness of the double layer 12. The Cdl is calculated from the Eq. (12) at the maximum frequency (fmax) 65:

**Table 6:** Electrochemical kinetic variables from EIS studies for C-steel in 1.0 M HCl without and with different doses of the studied inhibitors at 298 K.

Inhibitor	EIS data							
	Conc., M	R_ct_ Ω cm^2^	Y_o_,µΩ^−1^ s^*n*^ cm^−2^	n	C_dl_ μF cm^2^	Θ	%η_EIS_	Goodness of fit (χ^2^)
	Blank	30.6 ± 0.1753	306	0.849	161.4 ± 0.2122	–	–	15.71 × 10^–3^
MA-1440	5 × 10^–6^	107.5 ± 0.1664	211	0.853	113.1 ± 0.1612	0.715	71.5	17.44 × 10^–3^
	10 × 10^–6^	122.0 ± 0.2109	198	0.859	104.9 ± 0.1553	0.749	74.9	20.57 × 10^–3^
	15 × 10^–6^	135.5 ± 0.1632	187	0.859	97.2 ± 0.1762	0.774	77.4	19.11 × 10^–3^
	20 × 10^–6^	152.2 ± 0.1728	179	0.865	92.3 ± 0.1332	0.799	79.9	21.01 × 10^–3^
	25 × 10^–6^	170.9 ± 0.1543	170	0.874	88.2 ± 0.1751	0.821	82.1	16.33 × 10^–3^
	30 × 10^–6^	178.9 ± 0.2128	166	0.875	87.2 ± 0.2013	0.829	82.9	23.11 × 10^–3^
MA-1456	5 × 10^–6^	91.1 ± 0.1745	219	0.887	139.1 ± 0.1743	0.664	66.4	16.16 × 10^–3^
	10 × 10^–6^	99.1 ± 0.1453	214	0.888	131.6 ± 0.1451	0.691	69.1	13.61 × 10^–3^
	15 × 10^–6^	112.7 ± 0.1401	208	0.891	119.6 ± 0.1812	0.728	72.8	15.78 × 10^–3^
	20 × 10^–6^	127.6 ± 0.2024	193	0.896	108.0 ± 0.2109	0.760	76.0	18.87 × 10^–3^
	25 × 10^–6^	135.5 ± 0.1741	185	0.891	102.2 ± 0.2202	0.774	77.4	17.55 × 10^–3^
	30 × 10^–6^	141.1 ± 0.1224	180	0.899	98.8 ± 0.1913	0.783	78.3	16.22 × 10^–3^

12 $${C}_{dl}=\frac{1}{{2\pi f}_{max}{R}_{ct}}$$

**Figure 11:**
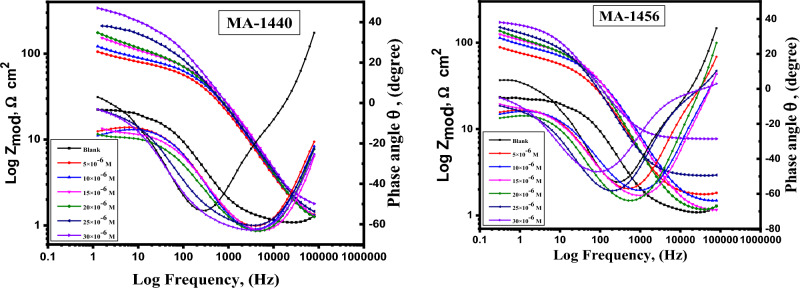
Bode graphs of the dissolution of C-steel in 1.0 M HCl with absence and presence of different doses of inhibitors at 298 K.

Bode absolute impedance plots, Fig. 11, shows a considerable increase in impedance magnitudes (*Z*_*mod*)_ with increased inhibitor concentrations over the whole frequency range, indicating an improvement in protection performance. The single peak that was shown in the Bode plots for both inhibitors demonstrated the existence of a single time constant, as indicated by the Nyquist plot. Additionally, significant values of the phase angle (ϴ) shown in the Bode phase plot confirm that greater inhibitory behaviour is obtained with an increase in inhibitor concentration^25^. Figure 10 makes it obvious that the experimental and theoretical curves fit together nicely. The evaluated chi-square **(χ**^2^**)** values **(**Table 6**)** suggest good fitting quality and the adoption of an equivalent circuit. The Table makes it very evident that the “n” value is closely correlated with the inhibitor concentration, whereas Y_o_ is the opposite. “n” is measurement of surface roughness^66^. In this work, an increase in the “n” value could indicate a decline in the electrode surface's heterogeneity as a result of the inhibitor molecules' adsorption. The obtained results from electrochemical measurements are compatible with those attained from *WL* experiments. The impedance outcomes confirm the inhibiting property of the investigated inhibitors obtained from *PDP* and *WL* measurements. The %IE of these derivatives is MA-1440 > MA-1456 as in PDP measurements.

### Surface examinations

#### Scanning-electron microscopy (SEM) analysis

**Figure 12:**
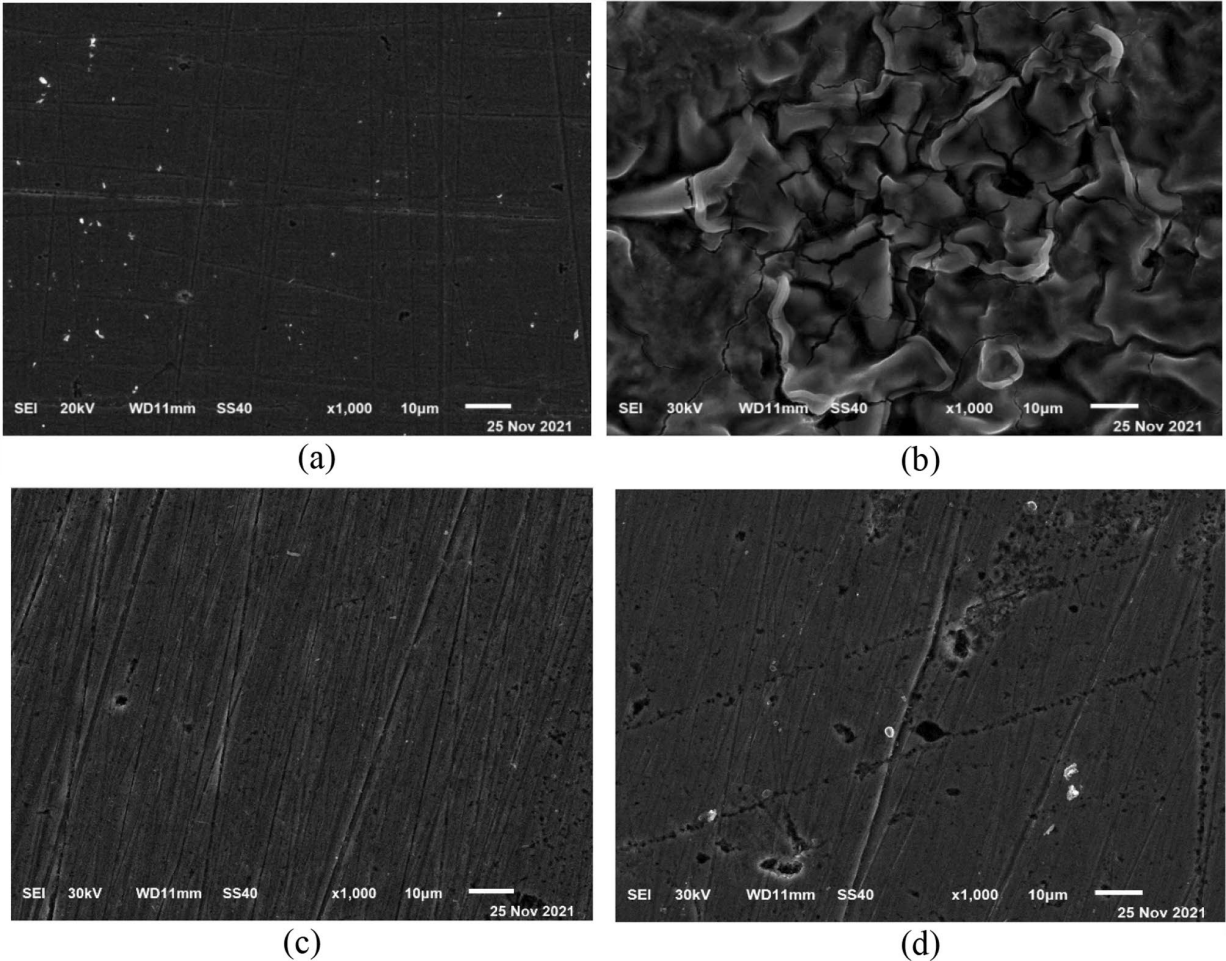
SEM images of: pure C-steel surface (**a**), after 6 h. dipping in 1.0 M HCl without inhibitors (**b**), in presence of inhibitor MA-1440 (**c**) and MA-1456 (**d**).

Using scanning electron micrographs, the surface morphology of carbon steel specimens was analysed before and after dipping in 1.0 M HCl for 6 h. with the presence and presence of inhibitors, at room temperature. The SEM pictures of C-steel taken before and after submerging in HCl are shown in Fig. 12a,b, respectively. As can be observed, the surface of C-steel before immersion in the solution is remarkably smooth compared to that after immersion in HCl without inhibitors, characterized by high destruction and extensive cracking consisting of dark spots covering the surface. This destruction is attributed to the aggressive attack of the acid solution on the exposed surface^29^. Figure 12c and d represent the scanned images of the retrieved coupons from the inhibitor-containing solutions. As expected, the inhibited samples exhibit comparatively smooth surfaces despite the presence of acidic medium. This can be explained on the basis that the presence of inhibitor in the solution depresses the rate of corrosion by creating a good shielding layer that protects the steel surface from the corrosive medium, reduces the surface roughness and preserves the surface morphology^67^. Moreover, observing carefully the images, the surface of the coupon inhibited by MA-1440 is relatively smoother than the one inhibited by MA-1456.

#### Energy-dispersive X-ray (EDX) studies

**Figure 13:**
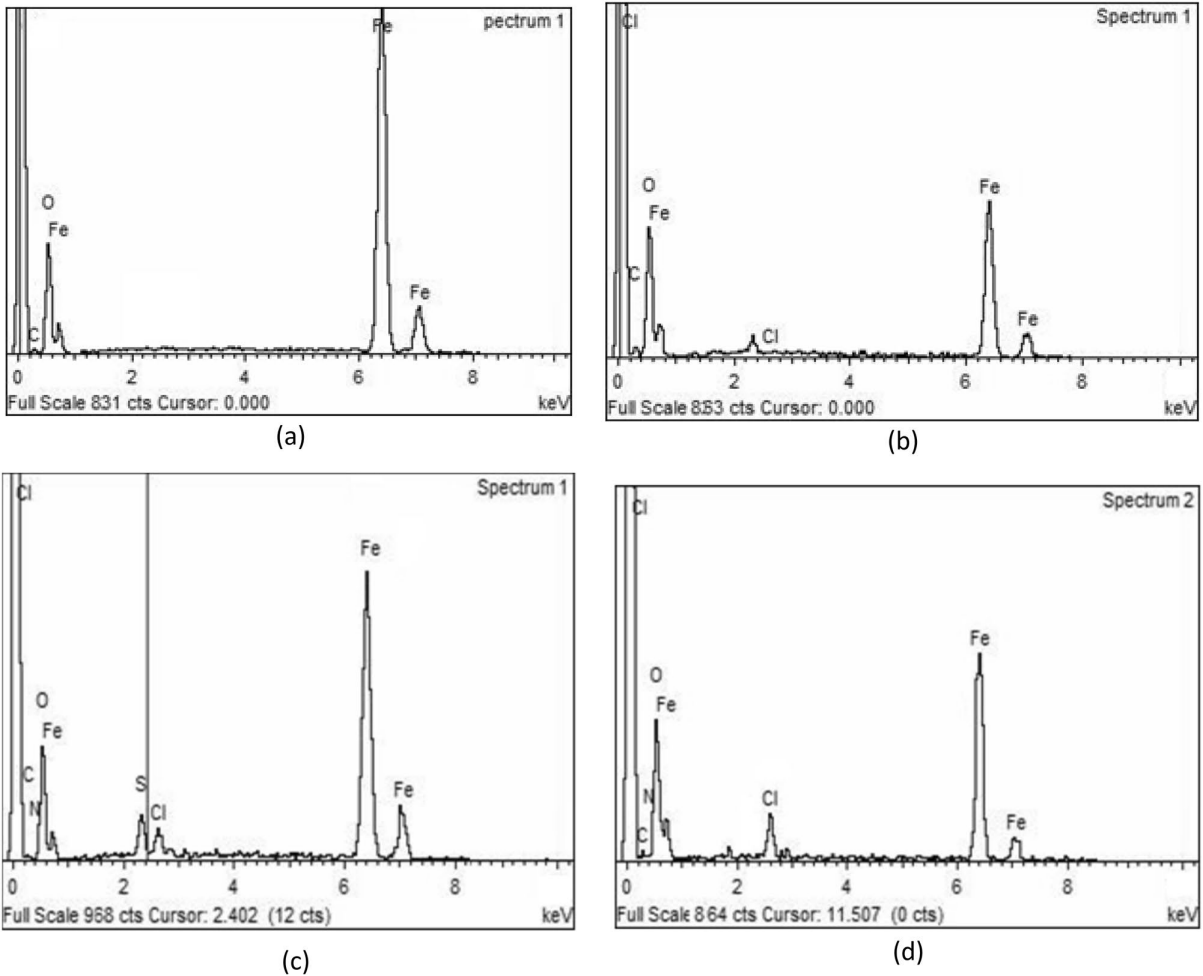
EDX spectra of C-steel surface (**a**), after 6 h dipping in 1.0 M HCl solution without inhibitors (**b**), in presence of 30 $\times$ 10^–6^ mol L^−1^ for MA-1440 (**c**) and MA-1456 (**d**).

Energy dispersive X-ray (EDX) investigation was utilized to examine the elemental constitution of carbon steel specimens as evidence of the adsorption of inhibitors on the steel surface. The related EDX spectra of carbon steel specimens are shown in Fig. 13. Table 7 lists the corresponding atomic concentrations of the detected elements for all samples. It is noticed that the iron content dramatically decreases in the sample exposed to the non-inhibited acidic solution compared with the non-exposed one. The oxygen content increases in corroded samples at the expense of iron owing to the creation of iron oxides (Fe_3_O_4_ and Fe_2_O_3_) on the steel surface as corrosion products^68^. On the other hand, the spectra of the samples retrieved from the inhibitor-contained solutions, exhibit an improvement in the content of iron which is proportional to the inhibition efficiency of the inhibitor. The more active inhibitor (MA-1440) showed as iron content higher than the inhibitor (MA-1456). The existence of nitrogen and/or sulphur in the spectra of the inhibited samples is a further confirmation of the adsorption of inhibitors on the steel surface. In conclusion, the creation of a shielding multilayer of inhibitors over the metal surface prevents the dissolution of iron in the medium and this technique is a further confirmation.

**Table 7:** EDX data obtained for C-steel surface analysis.

Samples	Weight %					
	Fe	O	C	N	S	Cl
C-steel	91.9	3.85	4.25	0.0	0.0	0.0
1.0 M HCl	60.23	29.28	8.31	0.0	0.0	2.18
MA-1440	68.97	11.95	11.7	2.38	2.21	2.79
MA-1456	65.06	16.76	10.21	3.41	0.0	4.56

#### Fourier-transform infrared spectroscopy (FT-IR) analysis

**Figure 14:**
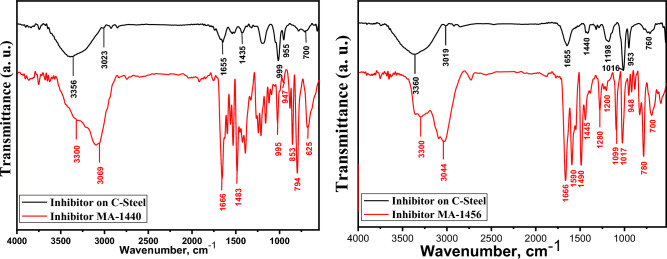
Comparison between IR spectra of pure inhibitors and C-steel surface after 6 h dipping in 1.0 M HCl solution with 30 $\times$ 10^–6^ mol L^−1^ of inhibitors at 298 K.

To demonstrate the interaction of the inhibitors with the carbon steel surface in 1.0 M HCl, infrared spectroscopy was performed. Figure 14 elucidates the comparison between the IR patterns of the pure solid inhibitors and the inhibitors adsorbed on the steel surface. By observing the IR spectra of the examined samples, the characteristics of the inhibited carbon steel coupons are identical in the pure inhibitors. The IR spectra of both studied inhibitors have characteristic and distinctive peaks. The inhibitor MA-1440 exhibits IR bands at 3300, 3069, 1666 and 1483 cm^−1^ characteristics of NH, CH, C=N, and C=C stretching vibration frequencies, respectively. A group of peaks are detected in the low frequency region at 625, 947 and 999 cm^−1^ ascribed to C–Fe, C–S and C–N, respectively^34^. The other IR spectra of the inhibitor MA-1456 exhibits four strong bands at 780, 1017, 1099 and 1280 cm^−1^ recognized as the symmetric and asymmetric vibration of the C–O–C group^69^, which are a characteristic of the bifuran fraction. The corresponding spectra of the inhibitors deposited over the carbon steel surface show positional fluctuation of IR absorption peaks as well as degradation of some peaks, indicating that the inhibitor molecules are well adsorbed over the steel surface. It is believed that the interaction between the heteroatoms in the inhibitors and the carbon steel surface is responsible for the shifts in the spectra. The coordination interactions between heteroatoms and Fe^2+^ result in the formation of Fe-inhibitor complex, which presents the protective coating over the active sites on the surface and increases the inhibition effect^70^. The low peak intensities in the spectra of the inhibited C-steel samples are explained considering the thin inhibitor layer covering the steel sample^62^.

#### X-ray photoelectron spectroscopy (XPS) analysis

**Figure 15:**
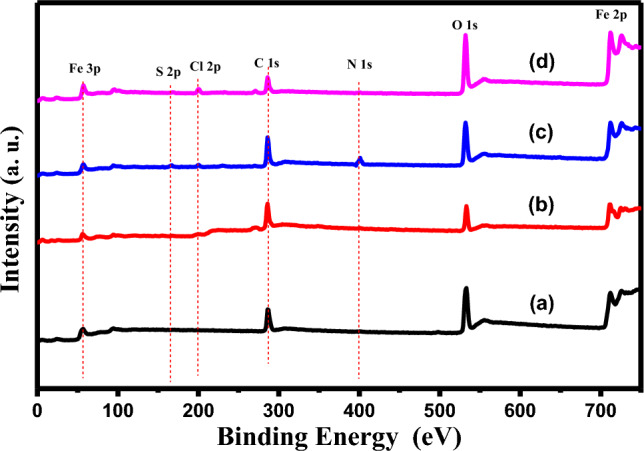
XPS survey spectra of (**a**) pure C-steel, (**b**) blank, (**c**) C-steel with MA-1440 inhibitor and (**d**) C-steel with MA-1456 inhibitor.

**Figure 16:**
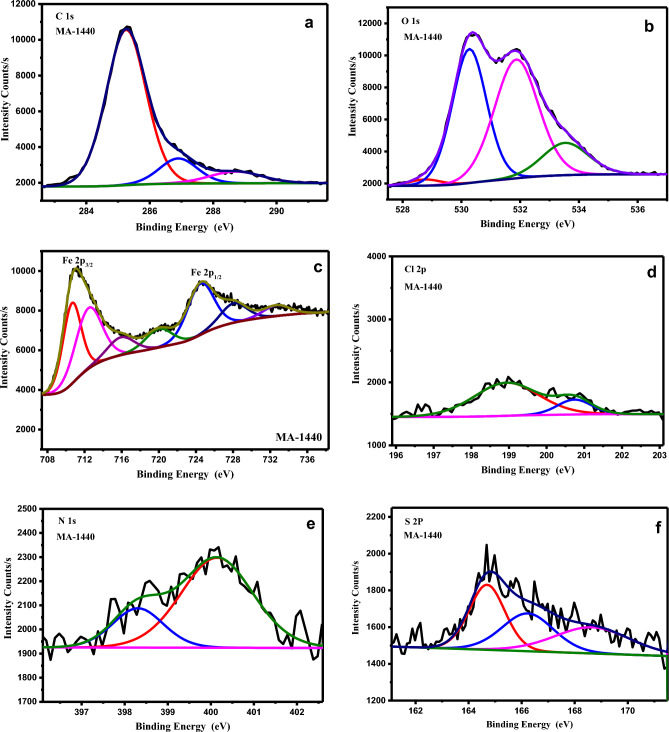
XPS deconvoluted profiles of (**a**) C 1*s*, (**b**) O 1*s*, (**c**) Fe 2*p*, (**d**) Cl 2*p*, (**e**) N 1*s* and (**f**) S 2*p* for the surface of C-steel dipped in MA-1440.

XPS analysis was performed to understand the nature of the chemical interaction at the inhibitor/carbon-steel interface and to analyse the elemental constitution of the deposited thin film on the carbon steel surface. Figure 15 displays the XPS survey of the samples retrieved after 6 h of immersion in 0.1 M HCl in the non-presence and presence of the optimized dose of inhibitors MA-1440 and MA-1456, in comparison with the pure C-steel sample. The spectra prove the presence of the elements involved in each sample. In particular, the deconvolution of the pure carbon steel spectrum shows C 1*s*, O 1*s*, and Fe 2*p* peaks. The spectra of specimens retrieved from the inhibitor-containing solution show the series of elements Fe 2*p*, O 1*s*, Cl 2*p*, C 1*s*, N 1*s*, and S 2*p* for the inhibitor MA-1440. While in the spectrum of MA-1456 the S 2p peak is missing according to the chemical structure of the inhibitor. As illustrated in Fig. 16a the spectrum of C 1*s* is deconvoluted and fitted into three distinct peaks. These peaks prove the existence of C in three chemical forms on the steel surface. The first intense peak positioned at 285.0 ± 0.15 eV refers to the C–H, C–C and C=C bonds^71^. The other two peaks at 286.9 ± 0.1 and 288.7 ± 0.2 eV refer to C–N and C=N, respectively^72,73^. Figure 16b displays the deconvolution of O 1*s* spectrum into three peaks located at 530.3, 531.8 and 533 eV. This peaks can be ascribed to oxygen atom bonded with Fe^3+^ as Fe_2_O_3_, hydrous iron oxides as FeOOH and adsorbed water, respectively^74,75^. As shown in Fig. 16c the spectrum of Fe 2*p* displays two profiles, Fe 2*p*_3/2_ and Fe 2*p*_1/2_ located at lower binding energy around 711 eV and at higher binding energy around 725 eV, respectively. The Fe 2*p*_3/2_ spectrum of pure carbon steel sample fitted into four peaks at 707, 708.7, 711 and 714 eV, attributed to metallic iron, Fe^+3^ as Fe_2_O_3_/Fe_3_O_4_, FeOOH and satellites of Fe^+3^, respectively^76^. Meanwhile, the deconvolution spectra of Fe 2*p*_3/2_ for the inhibited specimens involves three peaks at 711.3, 715, 719.5 eV ascribed to ferric oxide/hydroxide and, satellite of Fe^+2^ and Fe^+3^ compounds, respectively. Furthermore, the deconvolution of Fe 2*p*_1/2_ at higher binding energy can be assigned to the occurrence of iron oxides and hydroxide along with the contribution of Fe^+3^ in the inhibitor-Fe complex formation on the steel surface^77^. It should be noted that the peak at 707 eV of metallic iron disappears in the spectra of the inhibited specimens. This may be a result of inhibitor adsorption over the carbon steel surface which hence limits the ions diffusion and improves the corrosion inhibition of carbon steel in HCl solutions^78^. The deconvolution of Cl 2*p* (Fig. 16d) core-level fits with two components appearing at 200.5 and 199 ± 0.1 eV for Cl 2*p*_1/2_ and Cl 2*p*_3/2_, respectively. These peaks can be assigned to Cl–Fe bond in ferric chloride^79^. The presence of N 1*s* peak in the XPS analysis of the treated specimens can be used as the primary indicator of the adsorption of the examined molecules over the carbon steel surface. As shown in Fig. 16e, the spectra of N 1*s* are deconvoluted into distinctive double peaks. The first peak at 399.3 eV refers to the coordinated N–F bond, in which the nitrogen atoms of the inhibitor interacts co-ordinately with the empty d orbitals of the iron of the metal surface via donor acceptor interactions, resulting in the formation of an organometallic complex on the surface of carbon steel. The other peak at 400.5 eV is attributed to the N–C bond and =N– structure in inhibitor molecules^80^. The occurrence of nitrogen species on the inhibited specimens shows that, the examined molecules are adsorbed chemically on the surface of carbon steel. The spectrum of S 2*p* in Fig. 16f is observed only in the treated specimen with the inhibitor MA-1440. It is deconvoluted into three peaks at 164.15, 167.1 and 168.8 eV with two components, ascribed to C–S bond in bithiophene rings and coordinated S–Fe bond in the inhibitor-Fe complex^81^. The outcomes, based on XPS examination, show chemical interactions between the inhibitors under investigation and the surface of carbon steel. In fact, the existence of sulphur and nitrogen components on the steel surface, including C–S, =N– structure and N–C, as well as the formation of N–Fe and S–Fe bond complexes, suggests that the investigated inhibitors are adsorbed chemically on the carbon steel surface, creating a stable and uniform coating layer which protects the steel surface from the dissolution in acidic medium.

### Computational methods

#### Quantum chemical calculations

**Figure 17:**
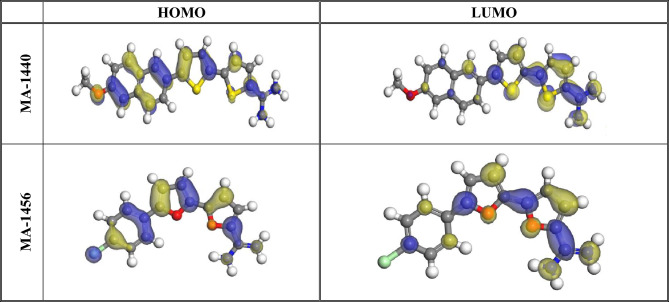
The highest-occupied (HOMO and lowest-unoccupied (LUMO) molecular orbitals calculated for the tested inhibitors at DMol3.

Quantum chemical computations were used as potential tools to investigate the inhibition mechanism of organic compounds in terms of their molecular and electronic structure characteristics. Figure 17 displays the molecular structure of the highest-occupied and lowest-unoccupied molecular orbitals, HOMO and LUMO, respectively. It is well known that the energy E_LUMO_ describes the ability to accept electrons, whereas the E_HOMO_ represents the ability to donate electrons. The ability of a molecule to donate electrons is directly related to HOMO energy (E_HOMO_) and higher is the HOMO energy, stronger is the tendency to donate electrons. On the other hand, the molecule with the greater negative value of LUMO energy has a stronger tendency to receive electrons^82^. Consequently, the capability of the adsorption of inhibitors on the substrate surface improves with high E_HOMO_ value and reduces with low E_LUMO_ value. It has the effect of increasing corrosion inhibition's productivity^83^. Similarly, the inhibition efficiency (%η) depends on the energy gap ΔE (ΔE = E_LUMO_ − E_HOMO_), being the higher inhibition efficiency associated with the lower energy gap value^42^. Based on Koopman's hypothesis, the HOMO and LUMO energies are related to the ionization potential (I_p_) and the electron affinity (E_A_), respectively, as depicted in the Eq. (13) ^80^:

13 $$Ip=-{E}_{HOMO}, {E}_{A}=-{E}_{LUMO}$$

The reactivity and stability of the molecules are associated also with other quantum-chemical parameters such as, absolute hardness (η), softness (σ) and global electronegativity (χ). These parameters are approximated according to following equtions^27^:

14 $${\varvec{\chi}}= \frac{{I}_{p }+ {E}_{A}}{2}$$

15 $$\eta = \frac{{I}_{P-}{E}_{A}}{2}$$

16 $$\sigma = \frac{1}{\eta }$$

The electrons fraction shared from the inhibitor molecule to the metal surface ΔN _(FET)_, is computed using the eqution (17)^25^:

17 $${\Delta N}_{(FET)}= \frac{{X}_{Fe}-{X}_{inh}}{2({\eta }_{Fe}+{\eta }_{inh})}$$

where *x*_*inh*_ and *η*_*inh*_ represent the absolute electronegativity and absolute hardness of inhibitor molecules, respectively. *X*_*Fe*_ and *η*_*Fe*_ indicate the absolute electronegativity and absolute hardness of iron. Table 8 reports the computed quantum-chemical parameters. The inhibitor MA-1440 has higher *ΔN*_*(FET)*_ and dipole moment (Debye) values than the inhibitor MA-1456. Moreover, the inhibitor MA-1440 has a lower energy gap and lower hardness than the inhibitor MA-1456. These results indicate that the inhibitor MA-1440 has the higher tendency to share electrons with the steel surface and consequently, the higher ability for adsorption on the steel surface and he higher inhibition efficiency. The theoretical calculations prove that the investigated inhibitors are adsorbed chemically on the steel surface. Additionally, the assumed theoretical hypotheses confirm and match with the experimental measurements.

**Table 8:** Quantum chemical statistics for the considered inhibitors.

Compound	E_HOMO_, eV	E_LUMO_, eV	ΔE, eV	I_P_, eV	E_A_, eV	η, eV	σ, eV	μ, eV	*∆*N	Dipole moment (Debye)
MA-1440	− 5.165	− 3.836	1.329	5.165	3.836	0.665	1.505	4.501	1.88	26.498
MA-1456	− 5.243	− 3.877	1.366	5.243	3.877	0.683	1.464	4.56	1.786	19.805

#### Monte Carlo (MC) simulation

**Figure 18:**
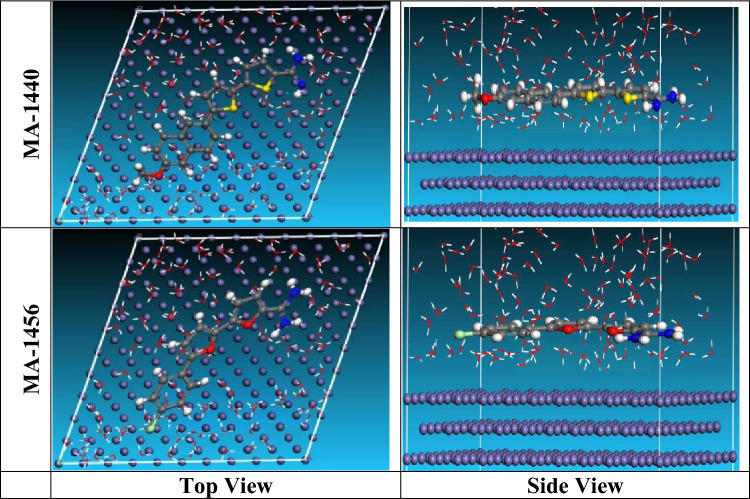
Monte Carlo simulation: top and side image of the perfect configuration for the adsorption of MA-1440 and MA-1456 inhibitors on Fe (110) surface.

Monte Carlo simulation tool provides a virtual modelling of the most stable and lowest energy configuration for the adsorption of organic molecules on the active substrates. Figure 18 Illustrates the top and side positions for the adsorbed inhibitors (M-1440 and MA-1456) on Fe (110)/50 H_2_O surface. It is evident from the side view images that both molecules are nearly parallel with closer contact to the Fe (110) surface, and appears lying flat on the surface of Fe (110) in the top view. This means an enlargement of the surface coverage of the substrate with the investigated molecules. Table 9 lists the derived parameters from the Monte Carlo simulation including the total energy of the substrate–adsorbate, in kJ mol^−1^. The adsorption energy is the required or liberated energy through the adsorption of the molecules on the substrate surface, and is equal the total of deformation’s and rigid’s energies^84^. As illustrated in the table, the adsorption energy for both inhibitors achieves high negative values, demonstrating the high adsorption stability of the inhibitors on the metal surface. Furthermore, the inhibitor MA-1440 displays lower adsorption energy than the inhibitor MA-1456 and this indicates its higher adsorption affinity and hence higher inhibition efficiency as found in experimental and instrumental techniques.

**Table 9:** Parameters calculated by the Monte Carlo simulation for adsorption of MA-1440 and MA-1456 on Fe (1 1 0).

Structures	Total energy kJ mol^−1^	Ads. energy kJ mol^−1^	Rigid Ads. Energy kJ mol^−1^	Deformation energy kJ mol^−1^	Compound dE_ad_/dNi	H_2_O dE_ad_/dNi
Fe (1 1 0) /MA-1440 /H_2_O	− 3101.6	− 3994.9	− 3925.778	− 69.125	− 276.389	− 11.652
Fe (1 1 0) /MA-1456 /H_2_O	− 3089.2	− 3990.68	− 3920.509	− 70.174	− 286.564	− 9.487

#### Inhibition mechanism of corrosion

**Figure 19:**
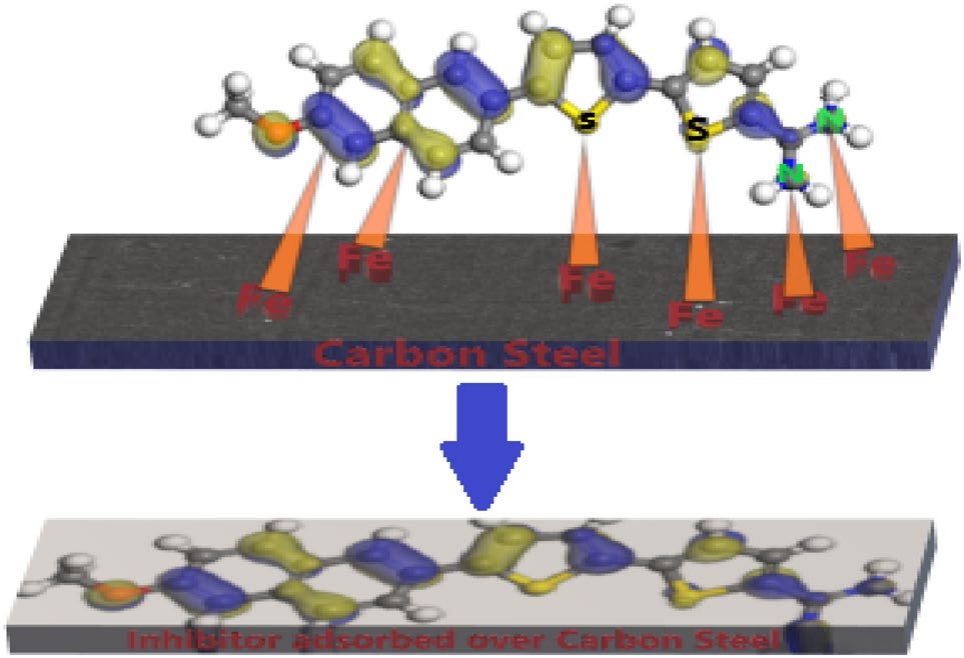
Graphic schematization of the adsorption mechanism of MA-1440 on C-steel in HCl solution.

The degree of inhibition may depend on a variety of parameters, including the reactivity of the substituents in aromatic rings, the electron density, the functional groups, the composition of the corrosive media, and the charges on the substrate surface^85^. According to the literature, adsorption can occur physically or chemically between the inhibitor and the C-steel surface. In the examined inhibitors, hetero atoms and aromatic rings are thought to be the mechanism's active centres. Figure 19 illustrates the possible mechanism of corrosion suppression considering the experimental and theoretical outcomes. As discussed, the investigated inhibitors are chemically adsorbed on the C-steel surface via donor–acceptor interaction and, this can be realized in two ways. The first way is accomplished through the electron transfer of the unshared pairs of electrons in π-orbitals of heterocyclic and aryl rings to the empty d-orbital in the iron atoms. The second way is achieved through the chemical coordination between the heteroatoms of the inhibitor as electron donors and the iron atoms as electron acceptor forming Fe-inhibitor complex. As a result of chemisorption interaction, metal-inhibitor complexes are created on the metal surface forming a protective layer that shields the C-steel from corrosion and additional metal dissolution. On the other hand, the protonated inhibitors in acid medium can also adsorbed on metal surface by interaction between them and the negatively charged metal surface (due to the adsorbed Cl^−^ ions on it) forming physical adsorption. The %IE of these derivatives is in the order: MA-1440 > MA-1456. This can be attributed to the difference in the molecular structures and the type of donating atoms, MA-1440 contains 2 S atoms that favourized the adsorption than O atoms present in MA-1456.

## Conclusion

1. In terms of the experimental and instrumental outcomes, the investigated inhibitors display excellent inhibition properties for the corrosion protection of C-steel in 0.1 M HCl at temperatures up to 328 K.
2. The inhibition efficiency increases with increasing the inhibitor dose in the solution, reaching the maximum at 30 × 10^−6^ mol L^−1^.
3. According to the thermodynamic studies, the inhibition efficiency of examined inhibitors is temperature-dependent and, the higher inhibition is achieved at the high temperature level.
4. The adsorption study reveals that the examined inhibitors obeyed Langmuir adsorption isotherm and are mainly chemically adsorbed on the C-steel surface.
5. Based on the Tafel polarization data, both inhibitors can be categorized as mixed inhibitors.
6. The EIS, PDP and WL measurements are in good agreement.
7. The instrumental examination using SEM, FT-IR, EDX, and XPS analysis confirms that the considered inhibitors are optimally adsorbed over the C-steel surface.
8. The theoretical investigation results are validated with those obtained by chemical and electrochemical methodologies.
9. Based on experimental and theoretical investigations, the inhibitor MA-1440 attains higher adsorption affinity and inhibition efficiency than the inhibitor MA-1456.

## Data Availability

The datasets generated and/or analysed during the current study are not publicly available due [REASON WHY DATA ARE NOT PUBLIC] but are available from the corresponding author on reasonable request.
